# Transient lupus anticoagulant induced by adenovirus cystitis in a bone marrow transplant recipient

**DOI:** 10.1007/s12185-025-04020-1

**Published:** 2025-06-05

**Authors:** Tatsuya Imi, Hidesaku Asakura, Keijiro Sato, Shinya Yamada, Takeshi Yoroidaka, Yoshitaka Zaimoku, Hiroyuki Maruyama, Kohei Hosokawa, Akiyo Yoshida, Hiroyuki Takamatsu, Ken Ishiyama, Hirohito Yamazaki, Hikaru Kobayashi, Shinji Nakao, Toshihiro Miyamoto

**Affiliations:** 1 Department of Hematology, Faculty of Medicine, Institute of Medical, Pharmaceutical, and Health Sciences, Kanazawa University, 13-1 Takaramachi, Kanazawa, Ishikawa 920-8640 Japan; 2 Department of Hematology, Nagano Red Cross Hospital, 5-22-1, Wakasato, Nagano, Nagano 380-8582 Japan

**Keywords:** Lupus anticoagulant, Hemorrhagic cystitis, Adenovirus, Transplantation

## Abstract

Adenovirus-associated hemorrhagic cystitis (AdV-HC) is a serious complication of hematopoietic stem cell transplantation (HSCT) that requires hemostatic therapies to control severe hematuria. Here we present the case of a 65-year-old woman with acute myeloid leukemia who successfully underwent HSCT, but developed AdV-HC followed by adenovirus viremia. Marked prolongation of activated partial thromboplastin time (APTT) was observed, along with a decrease in all coagulation factors except prothrombin, raising suspicion of a coagulation factor deficiency. A workup including the APTT cross-mixing test, diluted Russell’s viper venom time, and phospholipid neutralization assays revealed the presence of lupus anticoagulant (LA), indicating a thrombotic tendency due to LA associated with artifactual lowering of coagulation factors. Based on these findings, no hemostatic agents were used to manage macroscopic hematuria, and all coagulation test results spontaneously normalized with the resolution of adenovirus viremia, without any thrombus formation. Patients with AdV-HC and prolonged APTT should be screened for LA to avoid inappropriate use of hemostatic agents.

## Introduction

Anti-phospholipid antibody syndrome (APS) is an arterial and venous thrombotic disease caused by autoantibodies against phospholipids or proteins binding to phospholipids. Anti-phospholipid antibodies (aPLs) are sometimes detected after viral infections, but these are usually transient and disappear in several months [1]. Molecular mimicry, a phenomenon where foreign and host antigens share structural and functional similarities, leads to pathogen-specific immune responses that cross-react with self-antigens [2, 3]. Adenovirus (AdV)-associated transient aPLs with hypoprothrombinemia have only been reported in pediatric patients after adenoviral gastroenteritis or upper respiratory infections [4].

We encountered an adult patient with acute myeloid leukemia (AML) who developed AdV-associated hemorrhagic cystitis (AdV-HC) following allogeneic hematopoietic stem cell transplantation (HSCT). She presented with prolongation of the activated partial thromboplastin time (APTT) and a decrease in the level of several coagulation factors. The patient was initially suspected of having hemorrhagic coagulopathy, but was eventually diagnosed with a thrombotic tendency due to transient lupus anticoagulant (LA).

## Case presentation

A 65-year-old female underwent a bone marrow transplant from an HLA-one allele (HLA-DRB1) mismatched unrelated donor for the treatment of AML with *FLT3*-ITD while in complete remission (CR). The conditioning regimen included 180 mg/m^2^ of fludarabine, 9.6 mg/m^2^ of intravenous busulfan, and 2 Gy of total body irradiation (TBI). Graft-versus-host disease (GVHD) prophylaxis consisted of 2.5 mg/kg of rabbit anti-thymocyte globulin (ATG) on day-1 and continuous infusion of tacrolimus with short-term methotrexate (15 mg/m^2^ on day 1, 10 mg/m^2^ on days 3 and 6). Neutrophil engraftment and complete chimerism were achieved on days 15 and 20, respectively. The platelet count surpassed 20 and 50 × 10^9^/L on days 17 and 24 after bone marrow transplantation (BMT).

**Fig. 1:**
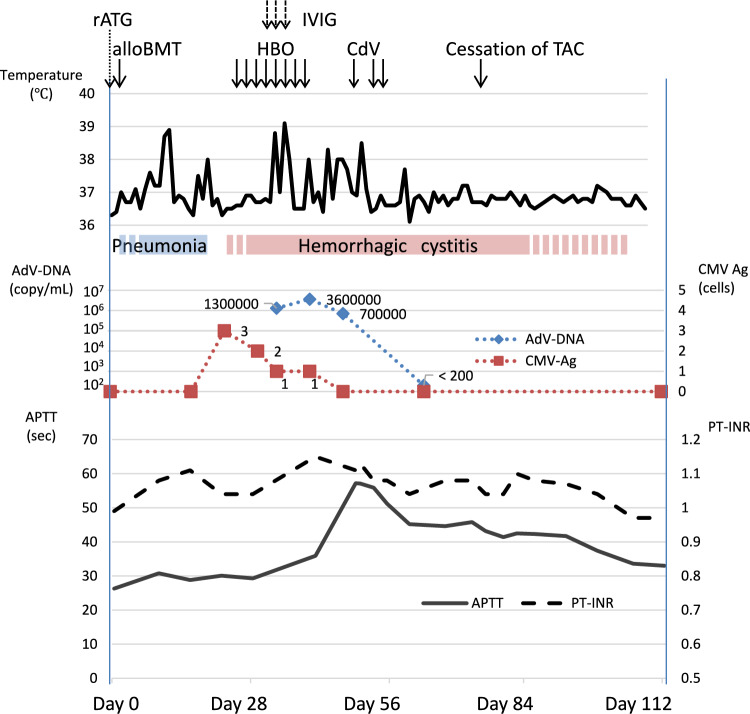
Clinical course of the present case. *rATG* rabbit anti-thymocyte globulin; *alloBMT* allogeneic bone marrow transplantation; *HBO* hyperbaric oxygen therapy; *TAC* tacrolimus; *CdV* cidofovir; *AdV* adenovirus; *CMV Ag* cytomegalovirus antigenemia

Microscopic hematuria began on day 17 after BMT, accompanied by frequent urination and micturition pain, and gradually progressed to persistent macrohematuria. AdV-HC was diagnosed because an AdV-specific polymerase chain reaction (PCR) assay of urine produced a positive result on day 24 (Fig. 1). In addition, ganciclovir was started as preemptive therapy for positive results of cytomegalovirus (CMV) antigenemia on day 24. Hyperbaric oxygen therapy (HBO) was started from day 28 to ameliorate AdV-HC, but her symptoms further deteriorated and became febrile. Her fever was unresponsive to acetaminophen and persisted, and high copy numbers of AdV-DNA in peripheral blood were revealed by a specific PCR assay on day 35, leading to a diagnosis of AdV viremia. Epstein–Barr virus DNA was negative. She manifested no sign of acute GVHD, leading to the tapering of tacrolimus. Intravenous immunoglobulin therapy (IVIG) at 0.01 g/kg/day for 5 days was initiated; however, the AdV-DNA copy number in the whole blood increased from 1.3 × 10^6^ on day 35 to 3.6 × 10^6^ copies/mL on day 42. Cidofovir (CdV) at a 1 mg/kg dose, administered thrice per week, was initiated as a clinical trial (Clinical trial number 6075 of Kanazawa University Hospital). She received it three times, but the fourth injection was canceled due to renal dysfunction. Her hematuria and fever rapidly resolved, along with a marked decrease in the AdV copy number (Fig. 1). Microscopic hematuria and urinary symptoms persisted but gradually disappeared following the cessation of tacrolimus on day 78.

**Fig. 2:**
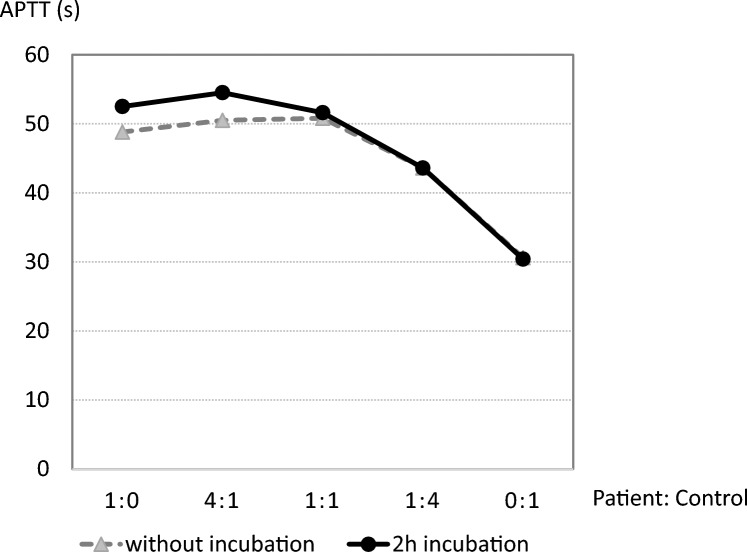
The results of cross-mixing tests without or with 2-h incubation. Cross-mixing tests were performed by measuring APTT in mixtures of control plasma with 100, 80, 50, 20, and 0% concentrations of patient plasma. The broken line with triangles and strait line with circles show the results for non-incubated and 2-h incubated mixtures, respectively

A prolongation of APTT (35.9 s; normal range: 27.3 to 40.3 s) was documented on day 45, when severe hematuria developed, and her APTT was further prolonged to 58.0 s by day 54. The patient did not have bleeding symptoms and physical manifestations except for hematuria. The international normalized ratio of the prothrombin time (PT-INR) remained normal. The levels of several coagulation factors including VIII (36%), IX (14%), XI (5%), and XII (11%) were low, while the factor II level was normal (88%). The level of von Willebrand factor was elevated (404%), reflecting the inflammatory state. The Bethesda assay detected factor VIII and IX inhibitors (FVIII-I and FIX-I) at low levels (2.0 and 2.0 Bethesda Units/mL, respectively). The immediate and delayed (two-hour incubation) type APTT cross-mixing test revealed the presence of lupus anticoagulant (LA, Fig. 2). The diluted Russell’s viper venom time (dRVVT) and phospholipid neutralization assays also indicated her plasma to be positive for LA. Anti-cardiolipin antibodies (aCL), anti-cardiolipin β2-glycoprotein I complex antibodies, and anti-phosphatidylserine-prothrombin antibodies (aPS/PT) were not detected. The chromogenic activity assays were used to retest coagulation factors VIII and IX, which were found to be 31.3% and 64.0%, respectively.

We, thus, attributed the prolongation of APTT to the presence of LA. Lowering of the coagulation factor levels as well as the detection of FVIII-I and FIX-I were considered to be factitious changes associated with LA. Whole-body computed tomography and ultrasonography of the lower extremity veins did not reveal any thrombus. The patient’s APTT started shortening after AdV viremia was resolved by the administration of CdV. Although macrohematuria disappeared, microhematuria and mild symptoms of dysuria persisted for over 100 days after BMT. APTT returned to normal by day 109 after BMT but aPL was still positive in the cross-mixing and dRVVT tests at last follow-up (Fig. 1).

## Discussion

AdV is a pathogen that causes gastroenteritis, bronchiolitis, pneumonia, and keratitis. Approximately 80% of children become serologically positive for AdV by the age of 5 [5]. The incidence of AdV infections, such as gastroenteritis and cystitis, in adult HSCT recipients is reported to range from 3 to 13.6% [6–8]. Our patient developed transient APTT prolongation attributed to LA associated with AdV viremia. Transient aPLs following AdV infections were previously observed in 18 pediatric patients with gastroenteritis (*n* = 12), upper respiratory bronchitis (*n* = 3), gastroenteritis and bronchitis (*n* = 2), and unknown infectious site (*n* = 1) [4, 9–15] as well as one young adult patient with upper and lower respiratory AdV infection during immunosuppressive therapy for severe asthma [16]. However, the mechanism underlying LA induction by AdV infection is unknown. Gharavi et al. reported that GDKV, a peptide that binds to phospholipids via β2GPI, binds to cardiolipin in vitro, and that immunization of mice induces aPL and anti-β2GPI antibodies in vivo, mimicking human β2GPI antibodies. They also reported that injection of anti-GDKV-derived monoclonal aPLs into mice promotes thrombus formation in vivo. They have shown that several synthetic peptides associated with CMV and AdV shared a structural similarity with GDKV and can induce aPLs through immunization against beta2-glycoprotein 1-like phospholipid-binding viral products [2, 3]. Among 19 patients with aPLs associated with AdV, the positivity rates of LA, aCL, and aPS/PT antibodies were 100% (19/19), 16% (2/12), and 91% (11/12), respectively (Table 1). The pathologic aPLs derived from AdV infection were strongly associated with aPS/PL, suggesting that aPLs may be induced by a cross-reaction between phospholipid-binding AdV products and PS-dependent PT. However, an alternative underlying pathogenesis may be involved because aPS/PT was negative in our case.

**Table 1:** The feature of anti-phospholipid antibodies previously found in patients with adenovirus infection

Age	Sex	Background	Bleeding symptoms	PT activity	Other decreased coagulation factor activity	LA	aCL	aCL-β2GPI or aβ2GPI	aPS/PT	Treatment	References
2	F	ND	Dental trouble	3%	ND	+	ND	NA	ND	Spontaneous recovery	Houbouyan L et al. Arch Fr Pediatr 1984
5	F	Gastroenteritis	Skin	26%	VIII, IX, XI, XII	+	–	–	+	ND	Amiral J et al. Thromb Res. 1997
5	F	Gastroenteritis	Knee swelling, dental trouble	11%	XII	+	–	NA	+	Spontaneous recovery	Jaeger U et al. Ann Hematol 1993
7	F	Gastroenteritis	–	22%	–	+	–	NA	+	Spontaneous recovery	Jaeger U et al. Ann Hematol 1993
4	F	Gastroenteritis, upper respiratory infection	–	10%	XII	+	–	NA	+	Spontaneous recovery	Jaeger U et al. Ann Hematol 1993
3	M	Upper respiratory infection	Skin, microhematuria	16%	XII	+	+	NA	+	Spontaneous recovery	Jaeger U et al. Ann Hematol 1993
4	F	Gastroenteritis	Skin	10%	–	+	NA	NA	NA	Spontaneous recovery	Male C et al. J Pediatr 1999
4	F	Upper respiratory infection	Skin	9%	IX, XII	+	NA	NA	NA	Spontaneous recovery	Male C et al. J Pediatr 1999
4	M	Gastroenteritis	Skin	18%	–	+	NA	NA	NA	Spontaneous recovery	Male C et al. J Pediatr 1999
8	M	Gastroenteritis	Skin	5%	–	+	NA	NA	NA	Spontaneous recovery	Male C et al. J Pediatr 1999
2	M	Gastroenteritis	Hemorrhagic diarrhea	16%	XII	+	NA	NA	NA	Spontaneous recovery	Male C et al. J Pediatr 1999
3	F	Gastroenteritis	Skin, nose, multiple haematomas	< 10%	VIII, IX, XII	+	–	–	+	Spontaneous recovery	Schmugge M et al. Eur J Pediatr 2001
10	F	Upper respiratory infection	Skin	65%	VIII, IX, X, XI	+	–	–	+	Spontaneous recovery	Mizumoto et al. Eur J Pediatr 2006
11	M	Gastroenteritis	Scrotal hematoma	Low	IX, XI, XII	+	–	NA	+	Vitamin K, FFP, IVIG	Knove K et al. Eur J Pediatr 2012
4	F	Gastroenteritis	Skin	Low	IX, XI	+	–	NA	NA	Steroids	Knove K et al. Eur J Pediatr 2012
3	F	Gastroenteritis	Skin, nose	Low	–	+	–	–	+	Spontaneous recovery	Shimizu T et al. Jpn J Clin Immunol 2014
5	F	Gastroenteritis	Skin	44%	–	+	NA	NA	+	Spontaneous recovery	Shimizu T et al. Jpn J Clin Immunol 2014
9	F	Gastroenteritis	Skin	48%	V, VIII, IX, X, XI, XII	+	+	+	+	Spontaneous recovery	Shimizu T et al. Jpn J Clin Immunol 2014
19	M	Upper respiratory infection, pneumonia	–	ND	NA	+	NA	NA	NA	Spontaneous recovery	Gopalakrishnan V et al. J Hosp Med 2016
65	F	Hemorrhagic cystitis	Macrohematuria	88%	VIII, IX, XI, XII	+	–	–	–	Spontaneous recovery	Present case

*LA* lupus anticoagulant; *aCL* anti-cardiolipin antibody; *aCL-β2GPI* anti-cardiolipin β2-Glycoprotein I complex antibody; *aβ2GPI* anti-β2-Glycoprotein I antibody; *aPS/PT* anti-phosphatidylserine-prothrombin antibody; *NA* not assessed; *ND* not described

Immune dysregulation early after HSCT may contribute to the induction of autoantibodies such as LA. Greeno et al. reported that LA was detected in 3% of BMT recipients and occurred more frequently in pediatric than adult recipients. In addition to viral infections including CMV and herpesviruses, the use of cyclosporine A or T-cell depletion for GVHD prophylaxis, and the use of busulfan/cyclophosphamide as a preparative regimen may influence the development of LA [17]. Two previous studies reported the transfer of aPLs from transplant sibling donors. One donor was diagnosed with systemic lupus erythematosus with APS, while the other donor was asymptomatic with LA and APTT prolongation. These transferred aPLs were detectable in the recipients and may have contributed to the cerebral thrombosis and catastrophic APS that developed 5 years after BMT [18, 19]. The use of busulfan and tacrolimus may have predisposed our patient to the development of LA.

The pathological significance of LA associated with viral infections remains unclear. Because hypoprothrombinemia occurs concurrently with transient aPLs in all pediatric patients with AdV infections, pediatric patients suffer from bleeding symptoms without thrombotic events, referred to as LA-hypoprothrombinemia syndrome. Our patient’s macrohematuria was initially suspected to be caused by the reduction in coagulation factor levels, but considering that her prolonged APTT resulted from aPLs, it was simply due to severe cystitis induced by AdV. Gopalakrishnan et al. reported that a 19-year-old male became positive for LA associated with APTT prolongation during treatment for severe AdV pneumonia but did not experience hemorrhagic or thrombotic complications [16].

It is possible that aPLs may have been transferred from the asymptomatic APS donor of immunoglobulin. The LA and dRVVT tests were positive even after 2 months, so the possibility of aPLs being a false positive due to transferred antibodies is low [20]. It is also possible that aPLs were produced as an immune response to immunoglobulin. However, since the therapeutic effect of IVIG has been confirmed in various autoimmune diseases, the possibility of new aPL occurring with this treatment is low [21].

In this case, aPLs were observed after HSCT, differing from the aPLs typically seen in pediatric or immunocompromised individuals with AdV infection. Recipient-derived plasma cells can persist for a couple of years after HSCT, and most antibody production relies on these recipient-derived plasma cells [22, 23]. Thus, the LA detected in this case might have originated from the recipient. Alternatively, antibodies might be produced by recipient-derived plasma cells due to a cross-immune reaction involving donor-derived T cells early after HSCT. It is also possible that the disappearance of aPLs was related to the transition from recipient-derived to donor-derived plasma cells.

LA-positive patients often exhibit low levels of several coagulation factors as well as FVIII and FIX inhibitors. These false results can be caused by LA that interferes with APTT-based factor activity assays and Bethesda assays to detect factor inhibitors [10–15, 24–31]. Such false-positive results mislead physicians into using hemostatic agents, such as tranexamic acid and activated coagulation factors, which can increase the risk of thrombosis [32–36]. Therefore, screening for LA is crucial when HSCT recipients complicated by AdV-HC present with prolongation of APTT. If LA is identified, hemostatic agents should be avoided in the management of hematuria.

## Data Availability

The datasets analyzed during the present study are not publicly available due to the need to protect patient privacy but are available from the corresponding author upon reasonable request.
